# Betaine- and L-Carnitine-Based Ionic Liquids as Solubilising and Stabilising Agents for the Formulation of Antimicrobial Eye Drops Containing Diacerein

**DOI:** 10.3390/ijms24032714

**Published:** 2023-02-01

**Authors:** Brunella Grassiri, Andrea Mezzetta, Giuseppantionio Maisetta, Chiara Migone, Angela Fabiano, Semih Esin, Lorenzo Guazzelli, Ylenia Zambito, Giovanna Batoni, Anna Maria Piras

**Affiliations:** 1 Department of Pharmacy, University of Pisa, 56126 Pisa, Italy; 2 Department of Translational Research and New Technologies in Medicine and Surgery, University of Pisa, 56126 Pisa, Italy; 3 Centre for Instrument Sharing of University of Pisa (CISUP), 56126 Pisa, Italy; 4 Research Centre for Nutraceutical and Healthy Foods “NUTRAFOOD”, University of Pisa, 56124 Pisa, Italy

**Keywords:** ionic liquids, ocular delivery, micelle, hydrotrope, bacterial keratitis, diacerein, rhein, eye drops, in vivo, carnitine, betaine, solubilising agent, stabilising agent, antimicrobial, anti-biofilm

## Abstract

The therapeutic efficacy of topically administered drugs, however powerful, is largely affected by their bioavailability and, thus, ultimately, on their aqueous solubility and stability. The aim of this study was to evaluate the use of ionic liquids (ILs) as functional excipients to solubilise, stabilise, and prolong the ocular residence time of diacerein (DIA) in eye drop formulations. DIA is a poorly soluble and unstable anthraquinone prodrug, rapidly hydrolysed to rhein (Rhe), for the treatment of osteoarthritis. DIA has recently been evaluated as an antimicrobial agent for bacterial keratitis. Two ILs based on natural zwitterionic compounds were investigated: L-carnitine C6 alkyl ester bromide (Carn6), and betaine C6 alkyl ester bromide (Bet6). The stabilising, solubilising, and mucoadhesive properties of ILs were investigated, as well as their cytotoxicity to the murine fibroblast BALB/3T3 clone A31 cell line. Two IL–DIA-based eye drop formulations were prepared, and their efficacy against both *Staphylococcus aureus* and *Pseudomonas aeruginosa* was determined. Finally, the eye drops were administered in vivo on New Zealand albino rabbits, testing their tolerability as well as their elimination and degradation kinetics. Both Bet6 and Carn6 have good potential as functional excipients, showing solubilising, stabilising, mucoadhesive, and antimicrobial properties; their in vitro cytotoxicity and in vivo ocular tolerability pave the way for their future use in ophthalmic applications.

## 1. Introduction

Ionic liquids (ILs) are organic salts characterised by a melting point below 100 °C [1,2]. Their tuneable features, in conjunction with peculiar properties such as their null vapour pressure, wide electrochemical window, and high thermal and chemical stability, address unmet needs in biomedicine, making ILs a subject of intense research in the last 20 years [3,4,5,6]. ILs are indeed already applied in chemical industries as green alternatives to organic solvents for their low volatility, non-flammability, and high stability under thermal and chemical stresses [6,7,8,9]. In recent years, a clear trend of using starting materials from renewable sources for the preparation of bio-based ILs has emerged [10,11,12]. In this regard, betaine and L-carnitine are two natural compounds that have been previously used in the preparation of deep eutectic solvents (DESs) [13]. There are more than 10^18^ possible combinations of ILs, and this implies the possibility of obtaining customisable materials simply by varying the combination of their anions and cations. ILs can be obtained with any desired property, such as good water solubility, increased drug absorption and dissolution, or the ability to target the active ingredient to the site of action [6,14]. Moreover, ILs have been the object of several studies for their antimicrobial properties [15] and, interestingly, bio-based betaine alkyl ester ILs have been studied as potential antimicrobials and herbicidal agents in combination with the iodo-sulphuron-methyl and dicamba anions [13,16,17]. On the other hand, l-carnitine alkyl ester ILs with different alkyl chain lengths were successfully used as hydrotropes and surfactants for the solubilisation of Disperse Red 13 and vanillin [18]. Based on the reported results, l-carnitine C6 atoms alkyl chain compound appeared particularly promising, thanks to its higher cytocompatibility compared to longer and shorter alkyl chain derivatives.

As the therapeutic efficacy of many topically administered drugs is strongly limited due to their low bioavailability [19], natural-based ILs could be exploited as functional excipients for ocular delivery of labile, non-soluble drugs.

**Figure 1:**
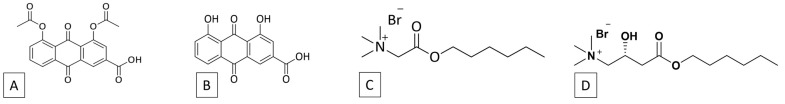
Chemical structures of active compounds and ILs applied in the study: (**A**) diacerein (DIA); (**B**) rhein (Rhe), the hydrolysed metabolite of DIA; (**C**) (2-hexyloxy-2-oxoethyl)-trimethylammonium bromide, Bet6 IL; (**D**) (R)-(4-hexyloxy-2-hydroxy-4-oxobutyl)-trimethylammonium bromide, Carn6 IL.

Diacerein (DIA, Figure 1A) is an example of a therapeutic that is not fully exploited, mainly because of its poor solubility and high instability. The poor solubility is due to its anthraquinoid structure. DIA is a slow-acting symptomatic prodrug for the treatment of osteoarthritis, authorised in countries of the European Union, Latin America, and Asia. Once administered, DIA is subjected to hydrolysis and is fully converted to its deacetylated active derivative rhein (Rhe) (Figure 1B). Several studies have proven other diverse therapeutic applications of DIA, e.g., as an anti-inflammatory agent in type 2 diabetes mellitus and psoriasis [20]; an antioxidant, hepatic, and nephroprotective agent [21]; an anticarcinogenic [22], and during the COVID-19 pandemic it was also evaluated for the treatment of SARS-CoV-2 [23]. Moreover, its antimicrobial properties have been pointed out, showing a bacteriostatic effect on *Helicobacter pylori* by inhibiting arylamine N-acetyltransferase [24]. Recently, the possible use of DIA in the treatment of bacterial keratitis has been investigated, opening the possibility for topical administration of the anthraquinone drug [25]. 

Bacterial keratitis is a corneal infectious disease caused by bacterial agents such as *Staphylococcus aureus*, *Pseudomonas aeruginosa, Streptococcus pneumoniae*, and *Serratia marcescens*. It causes symptoms such as sharp pain, redness, photophobia, tearing, blurred vision, and corneal ulceration, and can progress to blindness in severe untreated cases. Contact lenses, commonly used today, represent the biggest predisposing risk factor for infectious keratitis [26]. The risk is further increased for extended-wear (overnight-wear) soft contact lenses. The close interaction between the lens and the corneal epithelium induces local alterations, which affect the ability of the epithelium to respond to damage and limit the antimicrobial properties of tear fluid. In addition, contact lenses provide a surface where microorganisms may attach and colonise the surface as a biofilm [26].

Currently, both topical and oral antibiotic therapies are the treatments of choice for bacterial keratitis. Topically administered ocular drugs allow delivery of the active pharmaceutical ingredients (APIs) directly to their site of action, resulting in a reduction in the effective dose and in systemic side effects [19]. Although eye drops are currently the liquid formulation of choice for the topical treatment of ocular diseases, they are still very challenging from a drug delivery perspective. Indeed, blinking, basal tearing reflex, and nasolacrimal drainage limit drugs’ ocular residence time; as a result, frequent administrations are required [19]. Moreover, suspensions that are worse-tolerated cause increased mechanisms of corneal elimination; therefore, solubility plays a central role in drug bioavailability. 

The aim of the present work was to evaluate two C6 atom alkyl chain ILs, based on betaine and carnitine (Bet6 and Carn6; Figure 1C,D, respectively) as potential functional excipients with solubilising, stabilising, and mucoadhesive features. DIA was used as a poorly water-soluble, easily hydrolysable drug with potential antimicrobial features. Furthermore, Carn6 and Bet6 ILs were evaluated for their antibacterial properties against corneal keratitis pathogens, and the effects of their biopharmaceutical features were verified in vivo through the administration of IL/DIA-based eye drop formulations to rabbits.

## 2. Results

### 2.1. Determination of the Critical Aggregation Concentration (CAC)

**Figure 2:**
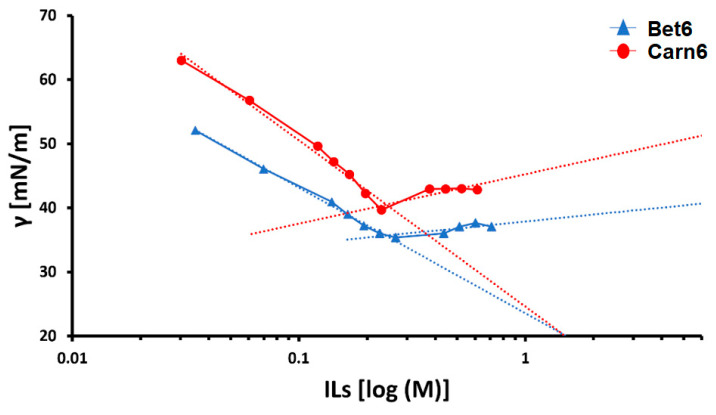
Determination of the critical aggregation concentrations (CACs) of a betaine-based ionic liquid (Bet6) and carnitine-based ionic liquid (Carn6), via surface tension. The CAC values of Bet6 (0.227 ± 0.016 M) and Carn6 (0.226 ± 0.020 M) are not statistically different (n = 3).

The CAC was evaluated by monitoring the surface tension values of the ILs’ aqueous solutions at different concentrations. Increasing the IL’s concentration caused a progressive decrease in the surface tension down to a limit value, in correspondence with the CAC. The CACs in water were 0.226 M (σ 36.1 mN/m) and 0.227 M (σ 34.2 mN/m) for Carn6 and Bet6, respectively (Figure 2). The data obtained in this study are consistent with those reported in the literature [18]. 

### 2.2. Dimensional and Zeta Potential Analysis 

The size distribution of the ILs’ aggregates, whether plain or drug-loaded, was determined using dynamic light scattering (DLS) measurements. Both ILs’ nanoaggregates showed an average diameter of ~2 nm in aqueous solution, suggesting that the different cations—betaine or carnitine esters—do not affect the size of the aggregates (Table 1). However, Carn6’s aggregates showed a slightly larger average diameter. With increasing concentration of the ILs, an increasing trend in the mean diameter was found, although this was not significant in the analysed concentration range. The addition of DIA to the IL solutions produced a small increase in the aggregates’ size. Aqueous solutions of the ILs displayed an acidic pH. The applied buffering conditions—citrate buffer pH 5 (CB) and phosphate buffer saline pH 7.4 (PB)—did not significant alter the nanoaggregates’ size distribution. The overlay of the representative diameter distributions of aqueous samples of nanoaggregates of Bet6 and Carn6, either alone or DIA-loaded, is reported in Figure S1 (Supplementary Materials). Both ILs are capable of interacting with DIA and with its hydrolysed active product Rhe, displaying a similar interaction trend with both substances in terms of nanoaggregate sizes and zeta potentials (Table 1). All of the analysed samples showed a positive surface charge, due to the presence of the ILs’ quaternary ammonium ion on the surface of the nanoaggregates. However, an effect of the buffers’ salt was observed. Indeed, in an aqueous non-buffered solution, both ionic liquids reduced the pH of the solution to about 4. In phosphate buffer (pH 7), a decrease in the net positive charge of the ionic liquids was observed. Conversely, switching to CB (pH 5) caused a drastic decrease in the zeta potential value. This was probably caused by the greater interaction of the citrate ion with the aggregates. The addition of drugs in aqueous solutions led to a reduction in the zeta potential. This effect could be attributed to the electrostatic interaction of both DIA and Rhe with the nanoaggregates of the ILs, thereby shielding the net positive charge of the ammonium ions. The zeta potential change was most evident for betaine-based ionic liquids, regardless of the presence of the drug. Since DIA was sensitive to degradation in PB, only Rhe medicated nanoaggregates were analysed in PB medium.

### 2.3. Evaluation of Bet6 and Carn6 as Solubilising Agents

**Figure 3:**
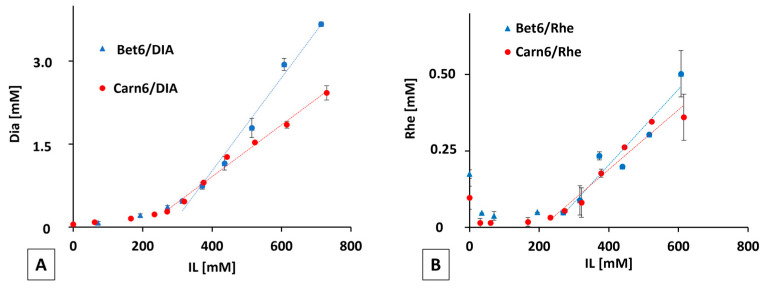
Evaluation of the apparent solubility of (**A**) DIA and (**B**) Rhe in the presence of ILs. Each point is the mean ± SD of three values.

DIA and Rhe have an anthraquinone scaffold that confers poor water solubility. Increasing the concentration of the ILs improved the apparent solubility of both DIA and Rhe. Notably, direct proportionality results were established at IL concentrations higher than the CAC (Figure 3A,B). Out of the two ILs, the betaine-based one had a greater solubilising power for both DIA and its active metabolite Rhe, although in the latter case the difference was not significant. 

### 2.4. Evaluation of Bet6 and Carn6 as Stabilisers

**Figure 4:**
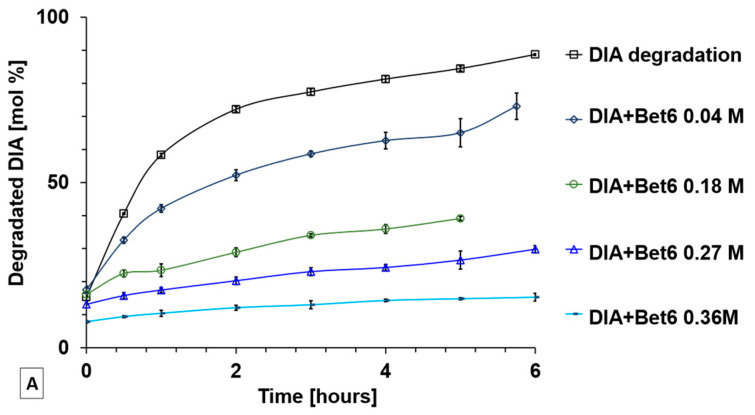

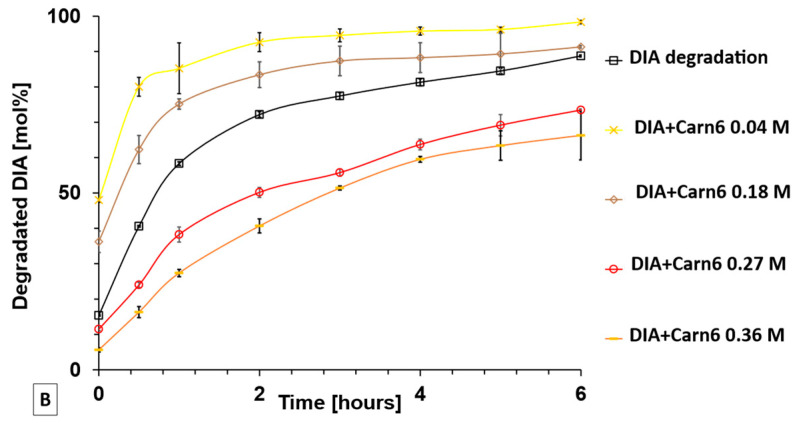
Effects of ILs in slowing down the degradation of DIA under simulated physiological conditions: (**A**) Bet6 solutions; (**B**) Carn6 solutions. Degradation of DIA is expressed in mol% and corresponds to the formation of Rhe. IL concentrations higher than 0.23 M (CAC) contain IL nanoaggregates. Each point is the mean ± SD of three values.

DIA is an insoluble molecule in water and at acidic pH, while its solubility increases at alkaline pH. However, in basic and neutral pH conditions, DIA is hydrolysed into its deacetylated derivative Rhe [27]. The hydrolytic degradation of DIA to Rhe in simulated physiological conditions was monitored at predetermined time intervals from 0 min to 6 h in the absence and presence of ionic liquids (Figure 4A,B). Furthermore, solutions of the ionic liquids were evaluated both in the absence and in the presence of nanoaggregates, corresponding to concentrations lower (i.e., 0.04 M and 0.18 M) and higher (i.e., 0.27 M and 0.35 M) than the CAC, respectively. In general, DIA’s rate of degradation was inversely related to the concentration of the Ils, and the Ils’ stabilising power became particularly efficient once exceeding the CAC. For Carn6, with 0.04 M and 0.018 M IL solutions, DIA was degraded by 98% and 91%, respectively. In these conditions, Carn6 did not form nanoaggregates and it was not effective in protecting DIA from degradation. Conversely, in the presence of Carn6 nanoaggregates, 26% and 34% of DIA was not hydrolysed after 6 h. 

The betaine-based IL stabilised DIA more effectively over time than the carnitine-based IL. Bet6 also slowed the degradation of DIA in the absence of nanoaggregates, reaching 73% and 39% for IL concentrations of 0.04 M and 0.018 M, respectively. Additionally, when Bet6 nanoaggregates were formed, they preserved DIA for 6 h, up to 71% and 85% in 0.27 M and 0.35 M IL solutions, respectively.

### 2.5. Evaluation of Mucoadhesion by Microrheological Method

The ILs’ mucoadhesion was assessed in vitro by monitoring the changes in the mucin solution’s rheology in the presence of different concentrations of ILs. The ILs were tested at concentrations 15% higher (0.27 M) and 15% lower (0.20 M) than the CAC. The results showed a substantial increase in the viscosity of the mucin solutions in the presence of both ionic liquids only when their concentrations were above the CAC, without finding any significant differences between the two ILs. (Table 2).

**Figure 5:**
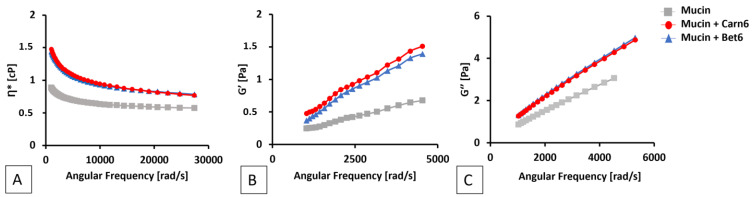
Rheological profiles of a mucin sample (reference) and mucin in the presence of ILs (either Bet6 or Carn6) at a concentration of 0.27 M (above CAC): (**A**) elastic modulus (G′); (**B**) viscous modulus (G″), (**C**) complex viscosity (η*).

The increase in complex viscosity was reflected in the increase in the moduli of G′ and G″ (Figure 5). At concentrations below the CAC, there was no increase in viscosity with respect to the mucin reference sample (data not shown). 

### 2.6. Cytotoxicity Evaluation

The murine embryonic connective tissue fibroblast cell line BALB/3T3 clone A31 (CCL-163) was used to determine the cytotoxicity of the ionic liquids, DIA, and Rhe after 4 h of incubation, according to ISO 10993-5 [28]. The 50% inhibitory concentration (IC_50_) of cell viability was determined, with results of 7.5 mg/mL and 9.0 mg/mL for Bet6 and Carn6, respectively. Concerning the active principles, the IC_50_ of DIA and Rhe was 116.2 µg/mL and 6.7 µg/mL, respectively. DIA had a lower cytotoxicity compared to its metabolite Rhe. The cytotoxicity profiles of the ILs and drugs are displayed in Figure S2 (Supplementary Materials).

### 2.7. Antibacterial and Anti-Biofilm Activity In Vitro

#### 2.7.1. Determination of MIC Values of DIA and Rhe 

The efficacy of the two anthraquinone compounds was tested on planktonic cells of *S. aureus* (clinical isolate W4) and *P. aeruginosa* (ATCC 27853 strain), representative of Gram-positive and Gram-negative bacterial species, respectively. DIA and Rhe both showed MIC values of 50 μg/mL against the clinical isolate *S. aureus* W4, whereas the MICs of both compounds against *P. aeruginosa* ATCC 27853 were higher than 50 μg/mL.

#### 2.7.2. Anti-Biofilm Activity of DIA and Rhe

**Figure 6:**
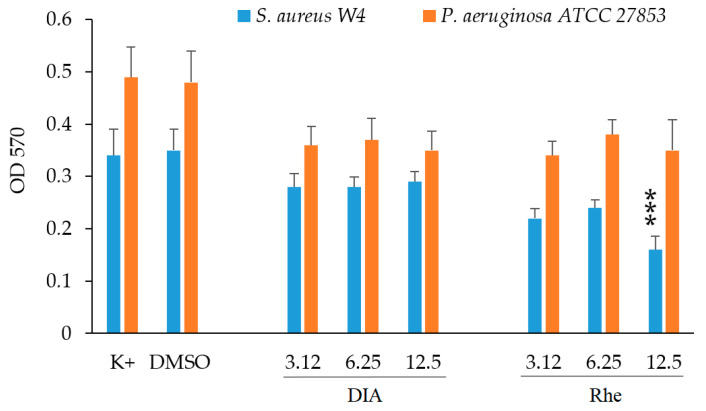
Anti-biofilm activity of DIA and Rhe against *S. aureus* W4 and *P. aeruginosa* ATCC 27853. Overnight bacterial cultures were diluted 1:100 and incubated with different concentrations of the two compounds. After 18 h of incubation at 37 °C, biofilm formation was quantified by CV staining. The figure depicts the mean values ± SEM of at least three independent experiments performed in duplicate. Concentrations are in μg/mL. Control (K+) represents bacteria incubated in medium only. DMSO represents bacteria incubated in medium with the addition of 0.8% DMSO; *** *p* < 0.001 (one-way analysis of variance (ANOVA) followed by the Tukey–Kramer post hoc test).

The ability of sub-inhibitory concentrations of DIA and Rhe to prevent biofilm formation by *S. aureus* and *P. aeruginosa* was tested by a standard microwell plate assay. Quantification of total biomass in the presence of different concentrations of each compound was evaluated by staining with crystal violet (CV)—a dye that is able to stain both bacterial cells and the biofilm’s extracellular matrix. After staining, CV bound in the biofilm biomass was solubilised and quantified by reading the optical density (OD) at 570 nm. When tested at 12.5 μg/mL against *S. aureus*, Rhe showed a statistically significant reduction in biofilm formation, whereas DIA showed no significant effects at any of the concentrations tested (Figure 6). Although a slight decrease in OD was observed when both DIA and Rhe were tested against *P. aeruginosa*, the differences were not statistically significant at any of the concentrations tested (Figure 6).

### 2.8. Eye Drops’ Formulation and Characterisation

To achieve both stabilising and solubilising effects, as well as mucoadhesive features, eye drop formulations were prepared by using the ILs at concentrations 15% higher than the CAC (i.e., at 0.27 M) to guarantee the presence of stable nanoaggregates. Despite the selected concentrations being approximately 10-fold higher than the in vitro IC_50_ values, the translation from in vitro to in vivo allows for a dose scaling. For example, benzalkonium chloride (Bak)—a cationic micelle-forming surfactant with bactericidal action—is applied in numerous marketed eye drops at concentrations up to 25-fold greater than its IC_50_ (Bak IC_50_: 9.4 μg/mL) [29,30].

Additionally, to guarantee DIA’s stability upon the eye drops’ preparation, CB pH 5 was used as a vehicle. The DIA concentration in the eye drops was set at 0.34 M, corresponding to the maximum value of apparent solubility that both ILs were capable of providing under the applied solution conditions. The compositions of the investigated eye drops were as follows:

- Solution of 0.34 M DIA and 0.27 M Bet6 (Bet6/DIA);
- Solution of 0.34 M DIA and 0.27 M Carn6 (Carn6/DIA);
- Suspension of 0.34 M DIA (DIA);
- CB pH5 used as a vehicle (CTRL).

#### 2.8.1. Antibacterial Activity of IL/DIA-Based Eye Drops

**Figure 7:**
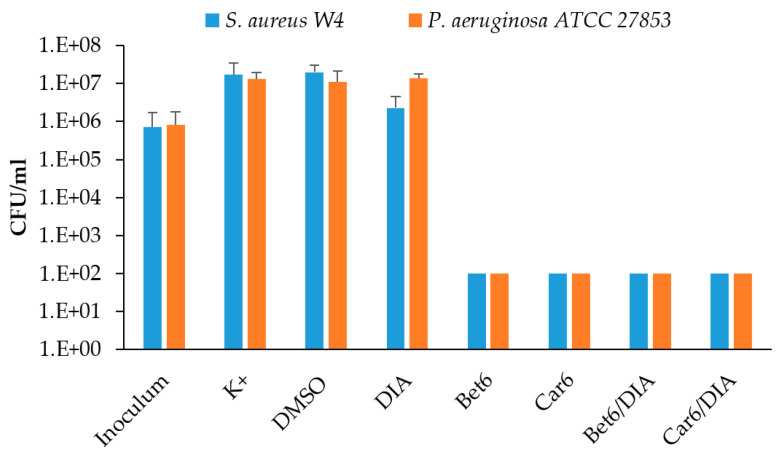
The antibacterial activity of the formulated eye drops based on DIA and ILs (Bet6/DIA and Car6/DIA) was tested against *S. aureus* W4 and *P. aeruginosa* ATCC 27853 after 3 h of incubation in 5% TSB. Unmedicated IL solutions (Bet6 and Car6) and a plain DIA suspension were also tested. K+ represents bacteria incubated in medium only. DMSO represents bacteria incubated in medium with the addition of 0.6% DMSO. The concentration of DIA used alone and with the ILs was 0.34 M, and the ILs were tested at 0.27 M. Bactericidal activity was defined as a reduction in the numbers of viable bacteria by ≥3-log colony-forming units (CFU)/mL after 3 h of incubation. Data are reported as the mean ± SEM of two independent experiments.

The antimicrobial properties of the formulated eye drops were evaluated in killing assays against *S. aureus* and *P. aeruginosa* after 3 h of incubation, and then compared to those of plain DIA, Bet6, and Car6 solutions. When DIA was tested alone, no evident bactericidal effect was observed against either *S. aureus* or *P. aeruginosa*. Conversely, Bet6 and Carn6, whether alone or loaded with DIA, showed a strong bactericidal effect against both microbial species, reducing the CFU below the limit of detection (100 CFU/mL) (Figure 7).

#### 2.8.2. In Vivo Studies

The prepared ocular formulations were tested in vivo in New Zealand albino rabbits by instillation of a single eye drop. 

##### Modified Draize Test

**Figure 8:**
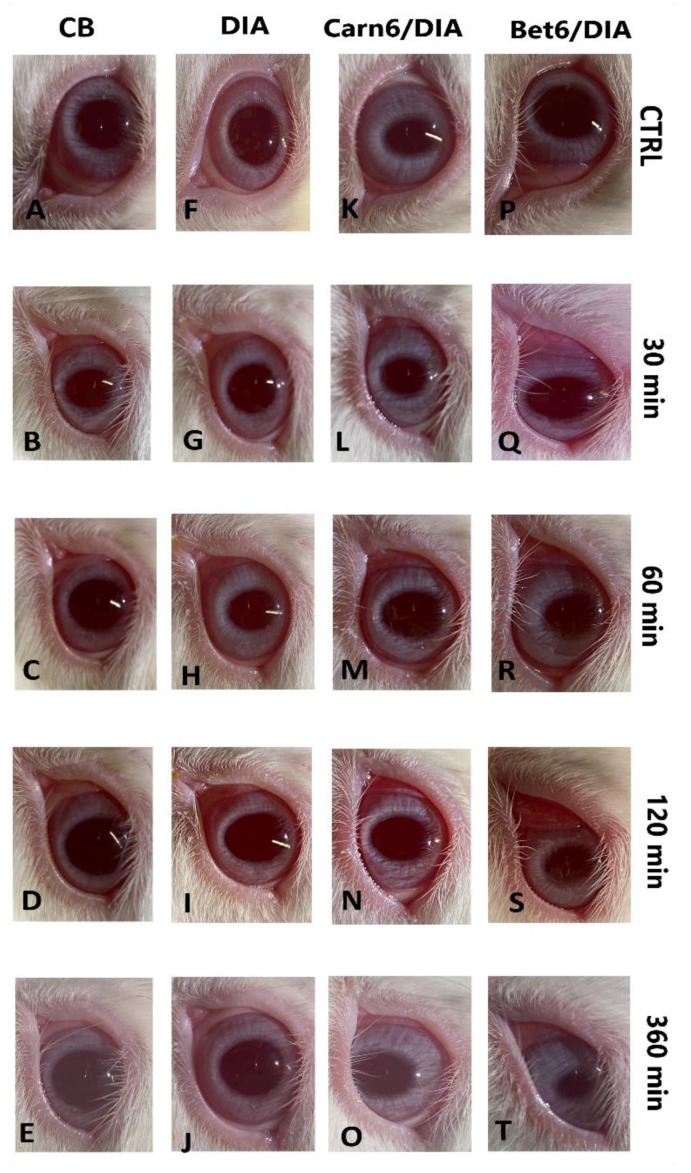
Modified Draize test representation: (**A**) Untreated right eye of rabbit CB used as a reference. (**B**–**E**) Left eye of rabbit “CB” after instillation of a drop of CB at 30 min, 1 h, 2 h, and 6 h, respectively. (**F**) Untreated right eye of rabbit “DIA” used as a reference. (**G**–**J**) Left eye of rabbit “DIA” after instillation of one drop of eye drops containing 0.34 M DIA at 30 min, 1 h, 2 h, and 6 h, respectively. (**K**) Untreated right eye of rabbit “Carn6/DIA” used as a reference. (**L**–**O**) Left eye of rabbit “Carn6/DIA” after instillation of one drop of eye drops containing 0.34 M DIA and 0.27 M Carn6 at 30 min, 1 h, 2 h, and 6 h, respectively. (**P**) Untreated right eye of rabbit “Bet6/DIA” used as a reference. (**Q**–**T**) Left eye of rabbit “Bet6/DIA” after instillation of a drop of eye drops containing 0.34 M DIA and 0.27 M Bet6 at 30 min, 1 h, 2 h, and 6 h, respectively.

A modified *Draize* test was applied to evaluate the irritancy to the eye caused by the eye drop instillation. The test (Figure 8) showed a modest redness of the conjunctiva and a slight conjunctival chemosis, but no secretion, during the first two hours after the application of the eye drops containing DIA, Bet6/DIA, or Carn6/DIA. No redness, secretions, or conjunctival oedema were detected for eye drops containing only DIA. The total I_irr_ score for each eye drop formulation was less than 3. Hence, it can be stated that all of the formulations are endowed with a good ocular tolerability.

##### Effect on DIA’s Stability and Retention on the Ocular Surface

**Figure 9:**
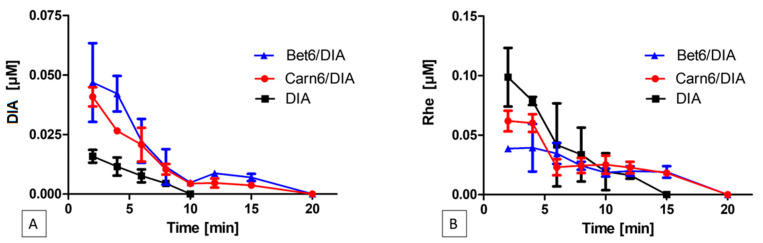
In vivo elimination kinetics from tear fluid assessed either (**A**) directly, by DIA monitoring, or (**B**) indirectly, by monitoring its hydrolysed active metabolite Rhe. Each point is the mean ± SD of 10 values.

DIA’s concentration in tear fluid (C_TF_) versus time profiles obtained with the different formulations were used to calculate the mean residence time (MRT) of DIA in tear fluid, according to the relevant non-compartmental theory [31]. MRT was determined from the AUMC/AUC ratio, i.e., the ratio between the area under the momentum curve, C_TF_*t vs. t (AUMC), and the area under the curve, C_TF_ vs. t (AUC) [32]. In addition to the MRT relating to each curve, Table 3 shows the maximum residence time of the drug at quantifiable concentrations in tear fluid (RT_max_). This time corresponds to the last point of the C_TF_ vs. time curve (Figure 9). Compared to the DIA suspension, the eye drops containing ILs provided improved results for all of the evaluated parameters. RT_max_ was 2.5-fold higher for both the Bet6 and Carn6 samples than for DIA alone, whereas the AUC was at least three times higher, with the Bet6 eye drops leading to statistically improved values. Furthermore, if the AUC and MRT obtained with the two ILs under study are compared, it can be observed that the values obtained with Bet6 eye drops are significantly greater than those for the Carn6 formulation. These results are consistent with those reported in Figure 4, where a greater ability of Bet6 to protect DIA from degradation can be observed. Moreover, it must be borne in mind that the tear fluid has a neutral pH and it is slightly buffered, representing a favourable environment for the hydrolysis of DIA to Rhe. 

As shown in Figure 9, for the eye drop formulation containing just DIA, the drug concentration rapidly dropped, indicating—as expected—a quick degradation of DIA (Figure 9A) to Rhe (Figure 9B). Conversely, for the IL-containing eye drops, the elimination of DIA from the tear fluid was slower, and its concentration remained higher than in the plain DIA eye drops at all time points.

## 3. Discussion

Bet6 and Carn6 are two bio-based ILs. Their behaviour in aqueous solution was evaluated, defining their solubilising and stabilising features toward DIA. ILs’ ability to act as surfactants is generally related to their hydrophobic alkyl chain length, because by increasing the length of the alkyl chains, the CAC decreases [18]. Furthermore, ILs with long alkyl chains show CACs in the order of the millimolar, behaving like common surfactants; in contrast, the CAC values of ILs with shorter chains more commonly fall in the molar order, typical of the minimum hydrotropic concentration (MHC) of hydrotropes [33]. The use of ILs as hydrotropes was first proposed in 2015 [14] and, although the mechanism by which hydrotropes are able to increase the water solubility of poorly soluble compounds is not yet fully understood, it has been hypothesised that for brominated carnitine esters this mechanism is comparable to micellar solubilisation [18]. The investigated Bet6 and Carn6 ILs had very similar CACs (0.227 M and 0.226 M, respectively), showing similar aggregation features, consistent with previous results collected on longer-chain derivatives [13]. The obtained nanoaggregates were uniformly distributed. Both types had an average diameter of 2 nm, confirming the hypothesis of Häckl et al. in 2018 [18] for carnitine ester derivatives. However, there was a tendency of Bet6 nanoaggregates to be smaller than those of Car6 at all tested conditions. Bet6 and Carn6 were able to efficiently increase the apparent solubility and stability of DIA. Both properties were more evident once the ILs’ concentration exceeded the CAC, consistent with a micelle-like solubilising effect. It is important to note that the increase in solubility was investigated by adding the drug to IL solutions without enhancing the aggregation phenomena that are sometimes observed in water/solute/hydrotrope systems [34]. It was also observed that the interactions of the nanoaggregates with both DIA and its metabolite Rhe caused only a slight increase in the nanoaggregates’ size but a significant reduction in the zeta potential value. This behaviour suggests a contribution to solubility due to the electrostatic interactions between the positively charged IL molecules and the negatively charged drugs, with the drug placed in the palisade layer of the nanoaggregates [35]. Additionally, increased apparent solubility corresponded to increased stability, with better performance by Bet6 nanoaggregates vs. Carn6, consistent with a micelle-like solubilisation of negatively charged drugs within positively shielded nanoaggregates [35]. In this context, it is worth highlighting the peculiar behaviour of Carn6. Indeed, an initial enhanced rate of DIA-to-Rhe conversion was found at low Carn6 concentrations, which was reversed once reaching the CAC. This different behaviour of Carn6 when compared to Bet6 should be related to the structural difference between the two ILs—namely, the additional hydroxyl group present in the former. It is therefore reasonable to assume that a transesterification reaction involving the free OH takes place at low Carn6 concentrations, consistent with what was observed in the carnitine shuttle system, which favours the degradation of DIA. Conversely, above the CAC, the hydroxyl groups of Carn6 must be confined within the nanoaggregates, and the overall behaviour resembles that of Bet6.

To better describe the potent solubilisation and stabilisation effects of the investigated ILs, their behaviours can be compared to that of HP-β-CD. Cyclodextrins are the most widely applied excipients displaying solubilising and stabilising properties [36,37]. Among these, HP-β-CD is already applied in ocular formulations [38]. Firstly, the apparent solubility enhancement of DIA in the presence of increasing concentrations of HP-β-CD was determined (Higuchi–Connors method, as previously reported [39]; Figure S3). The association constant of the formed complex (52 ± 0.87 M^−^¹) and the complexation efficiency (CE 0.002) were in accordance with the findings of a previously reported work [27]. The degradation study of DIA performed by using DIA/HP-β-CD complexes (1:3, 1:5, and 1:10 molar ratios) evidenced a poor stabilisation capability of all DIA/HP-β-CD solutions; no differences were noted for the 1:3 and 1:5 solutions with respect to the degradation of plain DIA, whereas 1:10 DIA/HP-β-CD slowed the hydrolysis, but 80% of DIA was already degraded after 3 h (Figure S4). Interestingly, whereas the solubilising properties of ILs before reaching the CAC are comparable to those of the HP-β-CD, they are dramatically better in the presence of nanoaggregates, in terms of both solubilisation and hydrolytic stabilisation features. The observed properties highlight the potential applications of Bet6 and Can6 in this field. 

The assembly of ILs into nanoaggregates is also necessary to observe a mucoadhesive effect, producing over twofold increases in the values of the complex viscosity, G′, and G″ of mucin samples. This effect is indicative of an interconnected microstructure between mucin and the assembled ionic liquids [40], indicative of a prolonged ocular residence time of the IL-containing eye drops and, thus, increased bioavailability [19].

Concerning the cytotoxicity, this has been a controversial topic for ILs, delaying their entry into the biomedical field [1,41]. It is possible to design ILs with low cytotoxicity, combining the structures of the cations, anions, and chain length [41]. In the case of Bet6 and Carn6, the two compared ILs only differed in the presence of the CH_2_-CH(OH)- moiety between the ester and the quaternary ammonium group, but with same aliphatic chain length and anion. Both displayed good cytocompatibility toward murine fibroblasts, with IC_50_ values of 7.5 mg/mL (26.8 mM) and 9.0 mg/mL (27.8 mM) for Bet6 and Carn6, respectively. These values are three orders of magnitude higher than that of the preservative Bak (predominant dodecyl alkyl derivative) and at least 10 times higher than those of the analogous monocationic/bromide ionic liquids of the alkylquinolinium series [30].

The in vivo kinetic study of DIA’s elimination in the tear fluid confirmed all of the in vitro results. In the modified *Draize* test, all formulations showed good ocular tolerability, despite the slightly acidic pH and high osmolality. Both IL-containing eye drop formulations were in the form of solutions; therefore, they showed better tolerability compared to a suspension, such as the plain DIA sample. Indeed, no redness, secretions, or conjunctival oedema were detected. These results, in line with those of the cytotoxicity tests, allow us to define both medicated eye drop formulations as suitable for ocular administration. 

The kinetic profiles of the IL-based eye drops showed a prolonged tear fluid residence time of DIA. In the absence of the ILs, DIA was no longer available on the ocular surface within eight minutes, due to both elimination and hydrolysis to Rhe. Conversely, for the IL-based formulations, both DIA and Rhe had an increased residence time in the precorneal area, resulting from the stabilisation and mucoadhesive effects of ILs. 

Finally, the IL-based eye drops exerted bactericidal effects against both *S. aureus* and *P. aeruginosa*—two microbial species that are often involved in ocular infections. The bactericidal action was surprisingly effective, exceeding that of plain DIA. Indeed, it was demonstrated that DIA and Rhe exert antibacterial effects against *S. aureus*, whereas they were inactive against *P. aeruginosa*, confirming the previously reported ability of both compounds to preferentially inhibit the growth of Gram-positive bacteria [25]. Both compounds belong to the anthraquinone class, determining their antibacterial activity mainly by inhibition/disruption of the redox processes within the bacteria [42]. Bacteria organised in biofilms display a dramatically reduced susceptibility (up to 1000-fold) to conventional antibiotics compared to their planktonic counterparts, causing a high rate of treatment failure and persistence of many types of infections (e.g., lung infections in cystic fibrosis patients, wound infections, biomaterial-associated infections) [43]. Similarly to other anthraquinones [44], Rhe—but not DIA—displayed the ability to inhibit the biofilm formation of *S. aureus* at sub-MIC concentrations, suggesting an anti-biofilm mechanism of action beyond the bactericidal effect. Therefore, a prolonged residence time of Rhe could provide further anti-biofilm activity to the bactericidal IL-based eye drops. Such combined prolonged treatment could represent a promising tool for the treatment of bacterial keratitis. 

## 4. Materials and Methods

### 4.1. Materials

Acetone ≥ 99.5%, acetonitrile (CH3CN) ≥ 99.5%, chloroform 99.0–99.4%, dimethyl sulfoxide (DMSO) ≥ 99%, HCl 1N standardised solution at 20 ° C, ethyl ether 99.8%, disodium phosphate dodecahydrate (Na_2_HPO_4_ ▪ 12H_2_O), methanol commercial product ≥ 99.8%, trisodium citrate dihydrate, 1-bromohexane, betaine, and carnitine were all purchased from Sigma-Aldrich, Merck KGaA, Darmstadt, Germany; TRIS (hydroxymethyl) e, monobasic sodium phosphate monohydrate (NaH_2_PO_4_ ▪ H_2_O), sulphuric acid 96% (H_2_SO_4_), and anhydride were purchased from Carlo Erba, Milan, Italy. Diacerein (DIA) ≥ 98% (MW: 368.29) and Rhein (Rhe) ≥ 98% (MW: 284.22) were acquired from Carbosynth, Biosynth AG, Switzerland, while hydroxypropyl-b-cyclodextrin (HP-β-CD) was acquired from Roquette.

### 4.2. Methods

#### 4.2.1. Synthesis of Betaine and L-Carnitine Ionic Liquids

(2-Hexyloxy-2-oxoethyl)-trimethylammonium bromide (Bet6) and (R)-(4-hexyloxy-2-hydroxy-4-oxobutyl)-trimethylammonium bromide (Carn6) were synthesised via O-alkylation reaction of betaine or l-carnitine with 1-bromohexane via a previously reported procedure [13]. 

(2-Hexyloxy-2-oxoethyl)-trimethylammonium bromide (Bet6): 98% yield, white solid; 1H NMR (D_2_O) δ 4.37 (s, 2H, NCH_2_COO), 4.31 (t, 2H, J = 6.6 Hz, CH_2_OCO), 3.37 (s, 9H, 3 × CH_3_N), 1.73 (quint, 2H, J = 6.5 Hz, CH_2_CH_2_COO), 1.41–1.30 (m, 6H, 3xCH_2_chain), 0.90 (t, 3H, J = 7.0 Hz, CH_3_chain) (Figure S5A); ^13^C NMR (D_2_O) δ 165.9 (COO), 68.1 (CH_2_OCO), 64.4 (NCH_2_COO), 54.8 (3 × NCH_3_), 31.3 (CH_2_chain), 28.2 (CH_2_chain), 25.4 (CH_2_chain), 22.6 (CH_2_chain), 14.0 (CH_3_chain) (Figure S5B). ^1^H spectrum was consistent with that reported in the literature [45].

(4-Hexyloxy-2-hydroxy-4-oxobutyl)-trimethylammonium bromide (Carn6): 92% yield, transparent viscous liquid; ^1^H NMR (D_2_O) δ 4.75–4.69 (m, ^1^H, CHOH), 4.26–4.15 (m, 2H, CH_2_OCO), 3.54–3.52 (m, 2H, NCH_2_), 3.27 (s, 9H, 3xNCH_3_), 2.77–2.65 (m, 2H, CHCH_2_COO), 1.73–1.66 (quint, 2H, J = 6.7 Hz, CH_2_CH_2_CH_2_COO), 1.42–1.31 (m, 6H, 3xCH_2_chain), 0.90 (t, 3H, J = 7.0 Hz, CH3chain) (Figure S6A); ^13^C NMR (D_2_O) δ 172.7 (COO), 70.0 (CH_2_N), 66.5 (CH_2_OCO), 63.2 (CHOH), 54.5 (3xCH_3_N), 40.6 (CH_2_COO), 31.0 (CH_2_chain), 28.1 (CH_2_chain), 25.1 (CH_2_chain), 22.2 (CH_2_chain), 13.6 (CH_3_chain) (Figure S6B). 1H and ^13^C NMR spectra were consistent with those reported in the literature [18].

#### 4.2.2. DIA and Rhe Quantification Methods

DIA and Rhe were detected and quantified by HPLC (series 200 pump, 20 μL loop, UV detector, and Turbochrom Navigator HPLC software for data integration; PerkinElmer, Waltham, MA). The Aeris 3.6 µm PEPTIDE XB-C18 column was applied, and the mobile phase consisted of 10 mM pH 5 methanol/citrate buffer (60:40) at 1.0 mL/min. Both DIA (4 min RT) and Rhe (9 min RT) were detected at l255 nm and quantified on 0.1–1 mg/mL standard curves (R^2^ = 0.987 and R^2^ = 0.994, respectively). 

As an alternative to HPLC determination, DIA and Rhe were also determined by UV–Vis spectroscopy. Briefly, UV–Vis spectra were acquired in the 200–600 nm range; DIA and Rhe were quantified at 368 nm and 472 nm, respectively, normalised at 600 nm. Quantification of the compounds was performed by using DIA and Rhe standard curves in the ranges of 10–100 µg/mL (R^2^ > 0.998) and 1–60 µg/mL (R^2^ > 0.997), respectively. 

#### 4.2.3. Characterisation of ILs’ Behaviour in Aqueous Solutions

##### Determination of the Critical Aggregation Concentrations (CACs) of the ILs

The CACs of the ILs were determined by measuring the surface tension (Tensiometer du Nouy, Force Tensiometer K6 from KRÜSS (GmbH)) of aqueous solutions with increasing IL concentrations. Milli-Q H_2_O was used as a reference, with γ equal to 71 ± 1.3 mN/m at 25 ± 2 °C (Dortmund databank reference, 71.5 ± 0.5 mN/m 24 ± 1.4 °C). Surface tension values were plotted vs. logarithms of the ILs’ concentrations, linear fits were applied to the data points, and CAC was determined as the intersection of the two linear fits relative to the initial surface tension depression and the further plateau [46]. 

##### Phase Solubility Study

The increase in the apparent solubility of both DIA and Rhe in the presence of the ILs was evaluated. Briefly, aqueous solutions of the ILs were prepared in the range of 0–200 mg/mL, and an excess of either DIA or Rhe was added to each vial. The samples were then incubated for 30 min at 25°C, and the undissolved drug was removed by centrifugation at 140,000 rpm for 15 min (Hettich Mikro 120 centrifuge). Notably, for DIA testing, 50 mM pH 5 citrate buffer (CB) was used to avoid DIA deacetylation. The drug concentration was measured by UV–Vis analysis. The DIA phase solubility study was also compared to the phase solubility study of DIA in the presence of HP-β-CD as a reference solubility/stability-enhancing excipient [27]. For HP-β-CD, a 1.5–15 mM concentration range was applied, comparable by weight with that used for ILs. The complexation K was calculated through Higuchi and Connors’ method, as previously reported [39].

##### ILs Aggregates’ Size and Zeta Potential in Aqueous Solutions

The size distribution and zeta potential values of the ILs’ aggregates in aqueous solutions were evaluated by using a Zetasizer Nano ZS (Malvern) at 25 °C. The samples were prepared by adding an excess of either DIA or Rhe (5 mg/mL) to the aqueous IL solutions, incubating them for 30 min at 25 °C, and finally removing the excess drugs by centrifugation. Citrate and phosphate buffers were used as reported in Table 1. DIA proved to be stable at pH values lower than 5; therefore, when CB samples were prepared, the drug and ILs were directly dissolved in the buffer. In contrast, for PB solutions, the solutions were prepared in water, and 10 mL of 100 mM pH 7 phosphate buffer was added per 1 mL of aqueous solution, directly in the cuvette, before the dynamic light scattering (DLS) analysis started. The concentrations of the solubilised drug were determined by UV–Vis analysis. 

##### Stabilisation of DIA by Using Ionic Liquids

The stabilising effect of Bet6 and Carn6 on DIA’s hydrolysis was investigated under simulated in vitro conditions. In detail, 4 mL solutions of 0.14 mM DIA, either alone or in the presence of ILs (0.03 M–0.3 M), were prepared in CB. After equilibrating for 30 min at 25 °C, the samples were transferred to a thermostatic shaking bath at 37 °C, and their pH was rapidly brought to 7.4 by adding 10 N NaOH. At defined time intervals (30 min, 1 h, 2 h, 3 h, 4 h, 5 h, and 6 h), a 500 µL sampling was performed, and the hydrolysis was blocked by adding 2 µL of 37% HCl, bringing the pH back to 5. The amount of Rhe formed over time was evaluated spectrophotometrically. Degradation of DIA was expressed in mol% of Rhe formation. The stabilising capability was compared to that obtained with HP-β-CD by applying the protocol to DIA/HP-β-CD solutions in CB, with molar ratios of 1:3, 1:5, and 1:10. 

#### 4.2.4. Microrheological Evaluation of ILs’ Viscoelastic Behaviour

The measurements were performed using the Zetasizer Nano ZS instrument, as previously reported [47]. The microrheological characterisation of the ILs was performed using type II porcine gastric mucin. Porcine gastric mucin was used because no ocular mucins were available, and as it has already been used for ocular models [48]. Briefly, a 3 mg/mL suspension of type II porcine gastric mucin was prepared in Milli-Q water and filtered through 0.45 µm cellulose acetate filters. Next, 0.27 M and 0.20 M solutions of both ionic liquids in deionised water were prepared (corresponding to concentrations 15% higher and lower than the CAC, respectively). One millilitre of each sample to be tested had the following composition: 5 µL of tracer (latex polystyrene particles, diameter 500 nm, Beckman), 7.38 mg/mL of IL solution, 0.3 mg of mucin, and 8.1 mg/mL of 5% NaCl. As a reference, 1 mL of sample was prepared with the same composition but without the ILs. All measurements were carried out in triplicate.

#### 4.2.5. Biological Assessments

##### Cell Culture Techniques

BALB/3T3 clone A31 fibroblast cells were grown in Dulbecco’s Modified Eagle Medium (DMEM), supplemented with 2 mM L-glutamine, 1% penicillin/streptomycin, and 10% bovine calf serum. Cells were grown in a CO_2_ incubator (Heracell 150i series) at 37 °C and 5% CO_2_, with cells sub-cultured at 80–90% confluency. The cell monolayers were rinsed with PBS and treated with trypsin–EDTA to detach the cells before their resuspension in fresh media. A subconfluent monolayer of BALB/3T3 clone A31 fibroblast cells was trypsinised, centrifuged at 1000 rpm for 5 min, resuspended in growth medium, and counted. 

##### Cytotoxicity Screening

A cytotoxicity screening was carried out for both ILs (range 0.05–25 mg), as well as DIA and Rhe (0.5–200 mg/mL). Briefly, BALB/3T3 clone A31 fibroblast cells were seeded in each well of 96-well plates at a seeding density of 6 × 10^3^ cells/well for 24 h, until 60–70% confluence was reached. After 24 h, the medium was then removed from each well and replaced with DMEM containing the samples.

After 4 h of incubation, the media were removed, replaced with fresh medium containing 10% WST-1 reagent solution, and maintained for 4 h at 37° C with 5% CO_2_. Afterwards, formazan dye absorbance was quantified at 450 nm with the reference wavelength at 655 nm by using a multilabel reader (BioTek 800/TS, Thermo Scientific).

#### 4.2.6. Microbiological Testing

##### Bacterial Strains

A clinical isolate of *S. aureus* (W4) and the reference strain of *P. aeruginosa* ATCC 27853 were used in the study. For the preparation of stock cultures, bacterial strains were grown in Luria–Bertani broth (LB, Oxoid, Basingstoke, Hampshire, UK) until the late-log phase, subdivided into aliquots, and kept frozen at −80 °C until use. Identification and susceptibility testing of the *S. aureus* W4 strain were performed using MALDI-TOF (Bruker Daltonics, Bremen, Germany) and the BD Phoenix susceptibility testing system (BD, Milan, Italy), respectively.

##### Determination of Minimal Inhibitory Concentrations (MICs)

The susceptibility of the bacterial strains to each of the tested compounds was assessed in terms of MIC values according to the standard microdilution method (Clinical and Laboratory Standards Institute–CLSI, 2018), with some modifications. Briefly, the bacteria were grown in LB broth until the exponential growth phase and then diluted in the same medium to reach a final density of 5 × 10^6^ CFU/mL. A volume of 10 μL of the bacterial suspensions was added to 90 μL of LB in a 96-well plate in the absence (viability control) or presence of the test compound at different concentrations. MIC values were defined as the lowest concentration of each compound resulting in the complete inhibition of visible growth after 24 h of incubation at 37 °C. 

##### Biofilm Inhibition Assay

After overnight growth in LB, cultures of *P. aeruginosa* ATCC 27853 and *S. aureus* W4 were diluted 1:100 in LB or in 50% tryptone soy broth (TSB, Oxoid) supplemented with 0.25% glucose (Sigma), respectively. The bacterial suspensions were dispensed into flat-bottomed polystyrene 96-well microplates (Corning Costar, Lowell, MA, USA) in the presence of sub-MIC concentrations of Rhe and DIA (3.12, 6.25, and 12.5 μg/mL). A sample containing 0.8 DMSO % (corresponding to the highest concentration of DMSO tested) was used as a control. The microplates were incubated statically at 37 °C for 24 h, and the biofilm biomass was estimated by CV (Sigma-Aldrich) staining assay, as previously described [49].

##### Killing Assays

The bactericidal activity of Bet6 and Carn6, alone or loaded with DIA (Bet6/DIA and Carn6/DIA), was evaluated against *S. aureus* W4 and *P. aeruginosa* ATCC 27853 in 5% TSB. To this end, the bacterial strains were grown in TSB until the exponential phase, and approximately 5 × 10^4^ CFU/mL—contained in 10 μL—was added to 5 μL of TSB, 35 μL of water, and 50 μL of one of the following solutions corresponding to the eye drop compositions tested in vivo: Bet6 or Carn6 with 0.27 M or 0.34 M DIA, plain DIA (0.34 M) solution, or plain Bet6/plain Carn6, both at 0.27 M. All solutions were prepared in CB. The samples were incubated at 37 °C with shaking for 3 h. A sample containing 0.6% DMSO (corresponding to the highest concentration of DMSO tested) was used as a control. Following incubation, the samples were diluted 10-fold in LB and plated on tryptone soy agar (TSA, Oxoid) to determine the number of CFU. Bactericidal activity was defined as a reduction of at least 3 Log10 in the number of viable bacteria as compared to the inoculum.

#### 4.2.7. In Vivo Studies

Male New Zealand albino rabbits weighing approximately 2.6–3 Kg, kept in standard housing conditions, were used for in vivo studies. The animals were treated as prescribed in the guidelines of the European Community Council Directive 2010/63. The animal protocol was approved by the Italian Ministry of University and Research (authorisation n. 192/2019-PR). The eye drops were prepared in CB (200 mOsm/kg). The ILs were added to a concentration of 0.27 M (above the CAC), and DIA was added for a final concentration of 0.34 M. The IL-based eye drops were named Bet6/DIA (645 mOsm/kg) and Carn6/DIA (588 mOsm/kg). A suspension of plain DIA (0.34 M) in CB was used as a reference sample (230 mOsm/kg). The samples’ osmolality was checked using a Roebling osmometer (calibrated with NaCl 300 mOsm/kg H_2_O). 

#### Draize Test

To assess the irritation of the rabbits’ eyes after instillation of the eye drops under study, a modified Draize test was performed, following the procedure previously described in [50]. This irritation test was conducted using a 0 (absence) to 3 (maximum) clinical rating scale for lachrymation, oedema, and conjunctival redness [51]. For each of the samples under study, the test was performed on three rabbits, through a single instillation of the eye drops (50 μL) per rabbit eye, while the untreated contralateral eye was used as a control. Each animal was observed at 0.5 h, 1 h, 2 h, and 6 h after instillation. An overall irritation index (Iirr) was calculated by adding the total clinical evaluation scores for each observation time. A score of 2 or 3 in any category or an Iirr greater than 4 was considered an indicator of clinically significant irritation.

#### Determination of DIA’s Elimination and Degradation Kinetics in the Tear Fluid

To determine the DIA’s elimination and degradation kinetics in the tear fluid, one drop of each sample under study (100 µL) was instilled with a Gilson pipette into the lower conjunctival sac of each rabbit eye, avoiding spillage. CB was used as blank control. At predefined intervals (i.e., 2, 4, 6, 8, 10, 15, 20, 25, and 30 min from instillation), tear fluid samples were taken from the lower margin strip using 1 µL disposable glass capillaries (Microcaps, Drummond Scientific Co., USA), which were washed with 1 µL and diluted with 100 µL of CB. The diluted samples were analysed by HPLC. Blank runs showed the absence of any interference with the measurements. For each of the three formulations studied, elimination curves were constructed, each determined from a single eye of different rabbits.

#### 4.2.8. Statistical Data Analyses

Each analytical test was conducted at least in triplicate on each batch sample. When possible, the datasets were statistically compared by applying Student’s *t*-test; *p* < 0.05 and *p* < 0.01 were considered indicative of a significant difference.

## 5. Conclusions

This work provides in vitro and in vivo evidence of betaine- and carnitine-based ILs as functional excipients capable of self-assembly into nanoaggregates, providing enhanced solubility and stability to DIA (a labile drug). The ILs were applied as excipients for ocular drug delivery, and both ILs exerted strong bactericidal activity on both *S. aureus* and *P. aeruginosa*—pathogens that are frequently involved in ocular keratitis. The formulated eye drops were able to solubilise the prodrug DIA, stabilise it, and increase its ocular residence time through mucoadhesion. Both in vitro and in vivo investigations indicated that the small structural difference between betaine and carnitine confers a significant difference in the solubilisation and stabilisation effect, with the betaine-based ionic liquid being more effective.

## Figures and Tables

**Table 1:** Main physical features of IL-based nanoaggregates: Aggregates size distribution and zeta potential of IL-based aggregates. Effects of concentration, pH, and drug solubilisation. Rhe and DIA were at saturation solubility for Bet6 solutions corresponding to 220 μg/mL and 1.350 mg/mL, respectively, and for Carn6 solutions corresponding to 130 μg/mL and 680 μg/mL, respectively.

Type	IL	DIA	Rhe	Medium	Ø	PDI± SD	ζ
of IL	(μg/mL)				(nm ± SD)		(mV± SD)
**Bet6**	90	-	-	H_2_O pH4	0.80 ± 0.03	0.01 ± 0.002	-
	105	-	-	H_2_O pH4	0.96 ±0.07	0.01 ± 0.005	-
	170	-	-	H_2_O pH4	1.96 ± 0.30	0.01 ± 0.005	-
	200	-	-	H_2_O pH4	1.81 ± 0.41	0.01 ± 0.005	13.70 ± 1.60
	200	-	+	H_2_O pH4	1.63 ± 0.21	0.01 ±0.005	8.17 ± 0.94
	200	+	-	H_2_O pH4	1.69 ± 0.92	0.02 ± 0.005	9.53 ± 1.68
	200	-	-	CB pH5	1.97 ± 0.44	0.03 ± 0.005	2.62 ± 0.96
	200	+	-	CB pH5	2.01 ± 0.60	0.02 ± 0.005	0.16 ± 1.85
	180	-	-	CB pH5	1.76 ± 0.38	0.01 ± 0.005	0.65 ± 0.53
	180	+	-	CB pH5	1.73 ± 0.40	0.01 ± 0.005	2.82 ± 1.17
	180	-	-	PB pH7	1.79 ± 0.41	0.01 ± 0.005	8.93 ± 0.94
	180	-	+	PB pH7	1.71 ± 0.49	0.03 ± 0.005	5.77 ± 0.54
**Carn6**	90	-	-	H_2_O pH4	1.17 ± 0.03	0.01 ± 0.002	-
	105	-	-	H_2_O pH4	1.53 ± 0.07	0.01 ± 0.002	-
	170	-	-	H_2_O pH4	2.12 ± 0.14	0.03 ± 0.016	-
	200	-	-	H_2_O pH4	1.87 ± 0.42	0.01 ± 0.005	14.30 ± 2.33
	200	-	+	H_2_O pH4	2.11 ± 0.50	0.02 ± 0.005	12.80 ± 1.27
	200	+	-	H_2_O pH4	2.14 ± 0.55	0.02 ± 0.005	12.20 ± 1.96
	200	-	-	CB pH5	2.34 ± 0.66	0.07 ± 0.005	2.08 ± 0.13
	200	+	-	CB pH5	2.40 ± 0.56	0.01 ± 0.005	4.17 ± 0.20
	180	-	-	CB pH5	2.04 ± 0.43	0.01 ± 0.005	2.56 ± 0.34
	180	+	-	CB pH5	2.24 ± 0.47	0.01 ± 0.005	1.85 ± 0.47
	180	-	-	PB pH7	1.91 ± 0.27	0.004± 0.005	10.70 ± 1.16
	180	-	+	PB pH7	2.03 ± 0.46	0.02 ± 0.005	9.38 ± 1.22

**Table 2:** Relative viscosity of mucin samples, either plain (mucin) or in the presence of Bet6 or Carn6 ionic liquids applied at 0.20 M (lower than CAC) or at 0.27 M (higher than CAC).

Sample	Relative Viscosity (cP)
Mucin	0.75 ± 0.03
Mucin/Bet6 (0.20 M)	0.79 ± 0.01
Mucin/Bet6 (0.27 M)	1.32 ± 0.13
Mucin/Carn6 (0.20 M)	0.75 ± 0.01
Mucin/Carn6 (0.27 M)	1.31 ± 0.26

**Table 3:** Effects of ILs on DIA residence time in the tear fluid of rabbits after instillation of one ophthalmic drop (50 μL) containing 0.34 M DIA and 0.27 M concentrations of different ILs. MRT, mean residence time; RT_max_, maximum residence time at measurable concentrations (≥2.5 μg/mL) (n = 10) (*** *p* < 0.0001 vs. DIA, ^++^ *p* < 0.005 vs. Carn6/DIA).

	DIA	Bet6/DIA	Carn6/DIA
**AUC**	0.06 ± 0.002	0.26 ± 0.006 ***^, ++^	0.19 ± 0.002 ***
**MRT (min)**	4.55 ± 0.03	6.43 ± 0.04 ***^, ++^	5.32 ± 0.02 ***
**RT_max_ (min)**	8	20	20

## Data Availability

Not applicable.

## Supplementary Materials

The following supporting information can be downloaded at: https://www.mdpi.com/article/10.3390/ijms24032714/s1, Figure S1: Diameter distribution by DLS analysis. Overlay of representative runs of aqueous samples of nanoaggregates of Bet6 and Carn6, at a concentration of 200mg/ml, either alone (Bet6; Carn6) or medicated with DIA (Bet6/DIA; Carn6/DIA). Figure S2: Cytotoxicity profiles of ILs (A) and drugs (B) on murine fibroblasts BALB/3T3 clone A31, after 4h of incubation. Figure S3: Comparison of Phase solubility studies of DIA in presence of ILs (Bet6 and Carn6) or HP-β-CD. Figure S4: stability study DIA alone or complexed with increasing concentrations of HP-β-CD. The tested concentrations were DIA/HP-β-CD molar ratio 1:3 (DIA1/HP-β-CD3); 1:5 (DIA1/HP-β-CD5); 1:10 (DIA1/HP-β-CD10). Figure S5. 1H NMR and 13C NMR spectra of Bet6. Figure S6. 1H NMR and 13C NMR spectra of Carn6.
