# Supplementary material for: Betaine- and L-Carnitine-Based Ionic Liquids as Solubilising and Stabilising Agents for the Formulation of Antimicrobial Eye Drops Containing Diacerein
Source: Int J Mol Sci. 2023 Feb 1;24(3):2714. doi: 10.3390/ijms24032714 (PMC9916883; doi:10.3390/ijms24032714)
Supplement: Supplementary file 1 [file ijms-24-02714-s001.zip › ijms-2174354-supplementary.pdf]

International Journal of
Molecular Sciences

MDPI

Supplementary material

Betaine- and L-Carnitine-Based Ionic Liquids as Solubilising and Stabilising Agents for the Formulation of Antimicrobial Eye Drops Containing Diacerein

Brunella Grassiri 1, Andrea Mezzetta 1, Giuseppantionio Maisetta 2, Chiara Migone 1, Angela Fabiano 1, Semih Esin 2,3, Lorenzo Guazzelli 1, Ylenia Zambito 1,4, Giovanna Batoni 2,3 and Anna Maria Piras 1,3,*

1 Department of Pharmacy, University of Pisa, 56126 Pisa, Italy
2 Department of Translational Research and New Technologies in Medicine and Surgery, University of Pisa, 56126 Pisa, Italy
3 Centre for Instrument Sharing of University of Pisa (CISUP), 56126 Pisa, Italy
4 Research Centre for Nutraceutical and Healthy Foods "NUTRAFOOD", University of Pisa, 56124 Pisa, Italy
* Correspondence: anna.piras@unipi.it

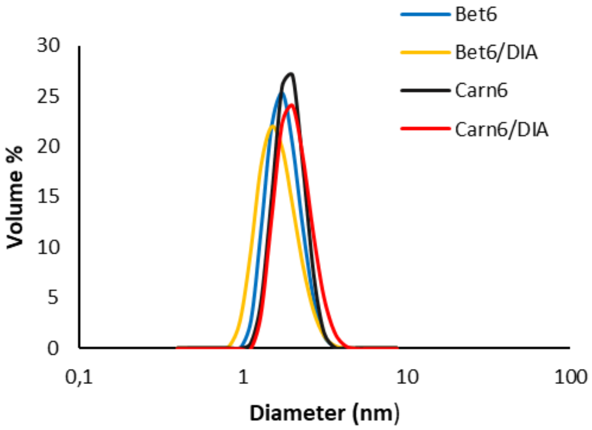

Figure S1. Diameter distribution by DLS analysis. Overlay of representative runs of aqueous samples of nanoaggregates of Bet6 and Carn6, at a concentration of 200mg/ml, either alone (Bet6; Carn6) or medicated with DIA (Bet6/DIA; Carn6/DIA).

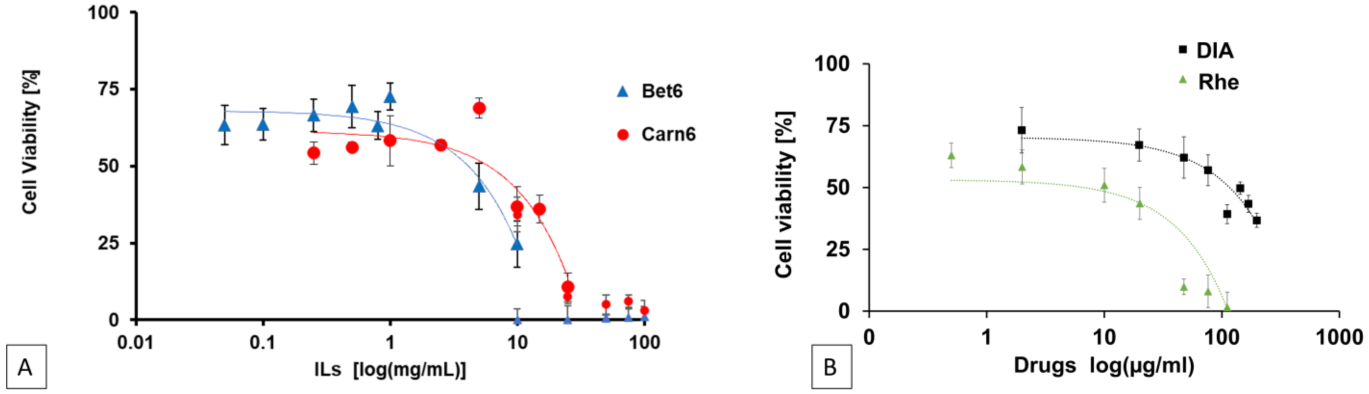

Figure S2. Cytotoxicity profiles of ILs (A) and drugs (B) on murine fibroblasts BALB/ 3T3 clone A31, after 4h of incubation.

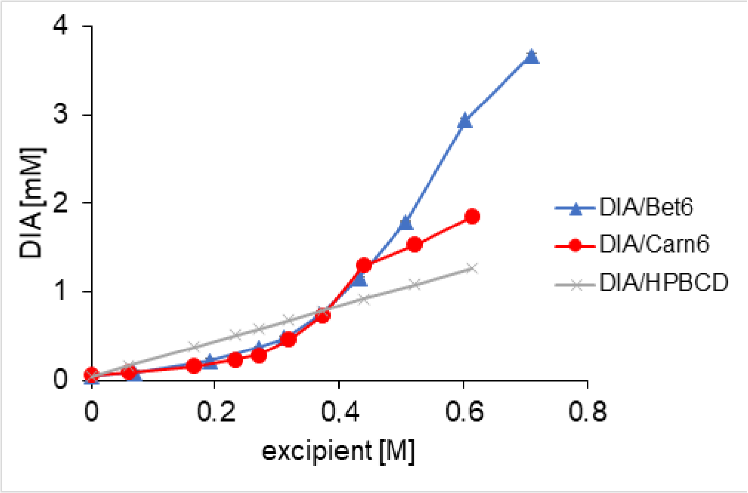

Figure S3. Comparison of Phase solubility studies of DIA in presence of ILs (Bet6 and Carn6) or HPβ-CD.

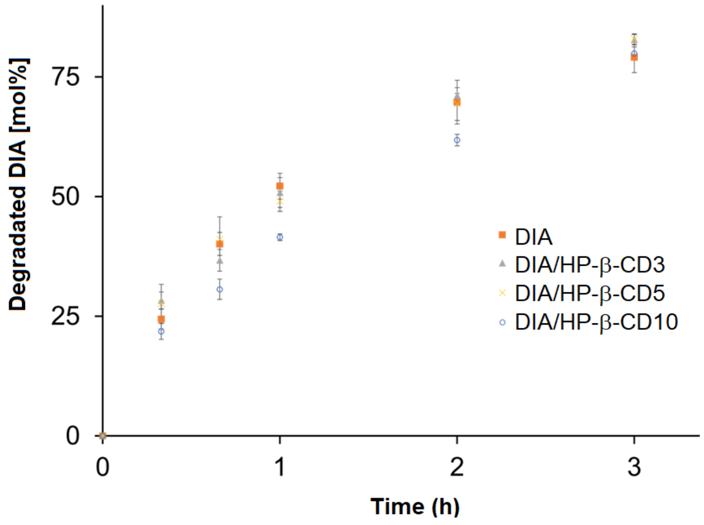

Figure S4. stability study DIA alone, or complexed with increasing concentrations of HP-β-CD. The tested concentrations were DIA/ HP-β-CD molar ratio 1:3 (DIA1/HP-β-CD3); 1:5 (DIA1/HP-β-CD5); 1:10 (DIA1/HP-β-CD10).

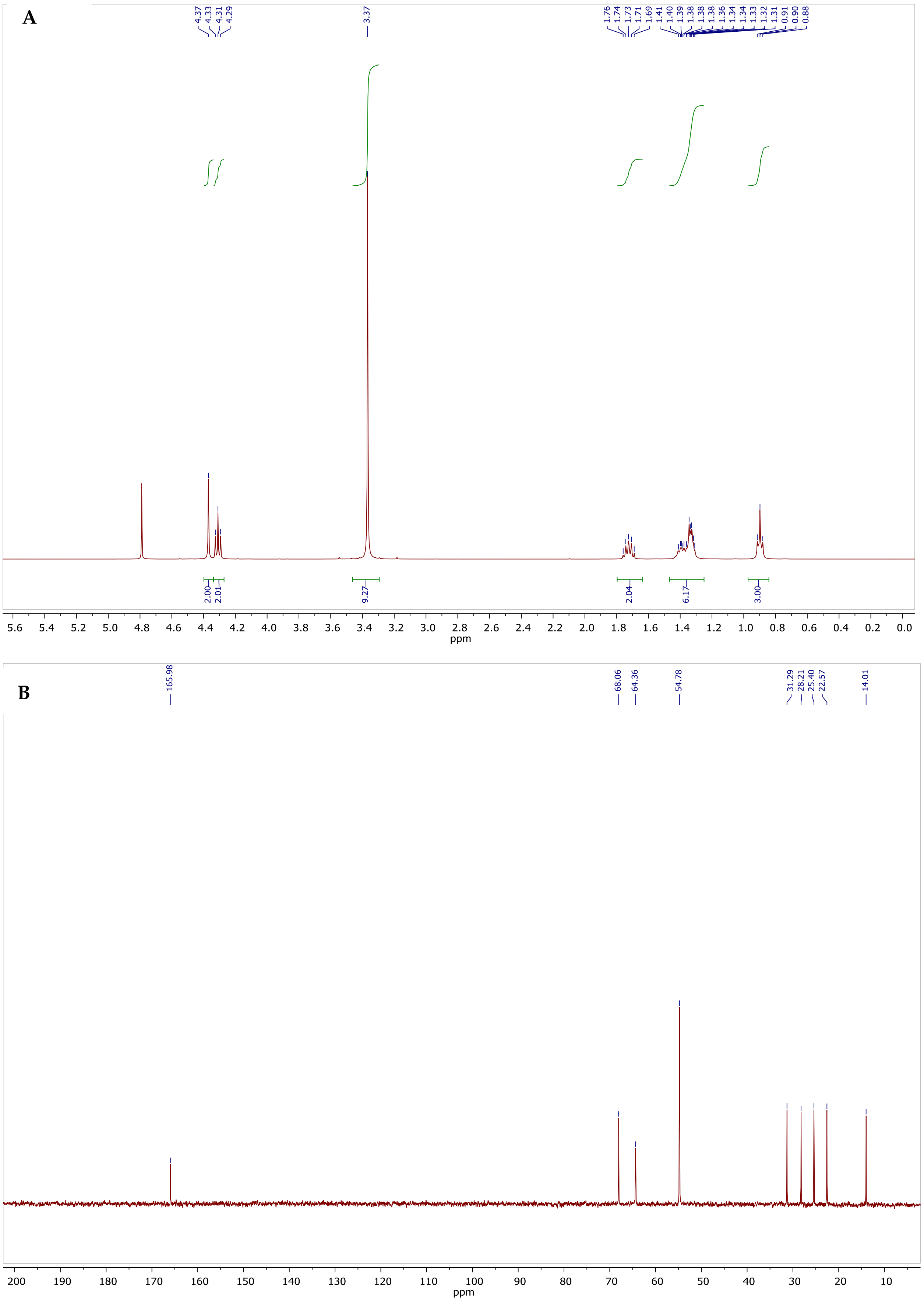

Figure S5. ^{1}H NMR and ^{13}C NMR spectra of **Bet6.**

A

B

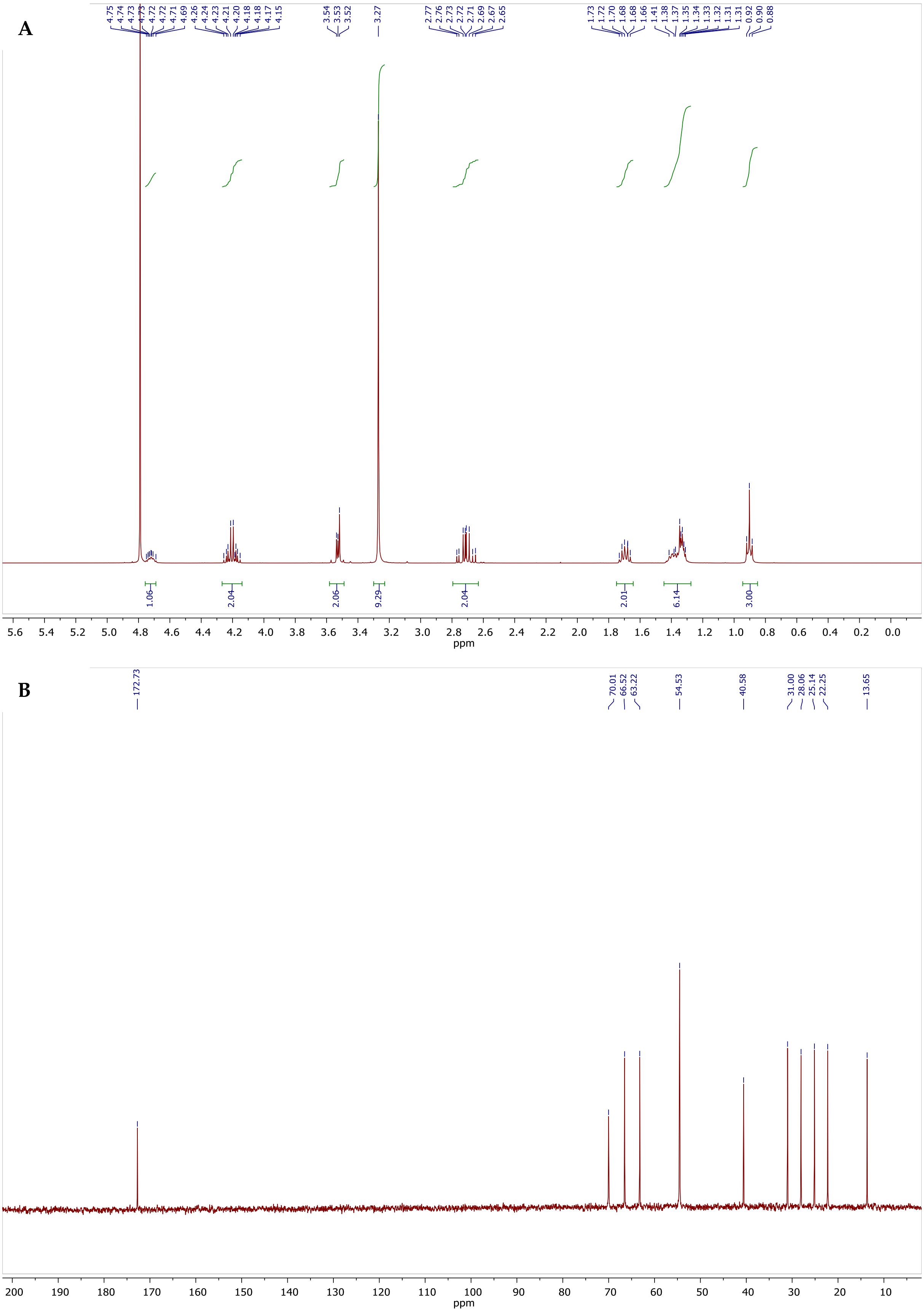

Figure S6. ^{1}H NMR and ^{13}C NMR spectra of **Carn6.**
